# Supplementary material for: Perceived stress and well-being of Polish migrants in the UK after Brexit vote
Source: PLoS One. 2020 Jul 23;15(7):e0236168. doi: 10.1371/journal.pone.0236168 (PMC7377441; doi:10.1371/journal.pone.0236168)
Supplement: Attachment — Submitted filename: Response to Reviewers.docx [file pone.0236168.s001.docx]

Thank you for submitting your manuscript to PLOS ONE. After careful consideration, we feel that it has merit but does not fully meet PLOS ONE’s publication criteria as it currently stands. Therefore, we invite you to submit a revised version of the manuscript that addresses the points raised during the review process.

Your paper has been assessed by two acknowledged experts in the area covered by this study. Overall, you will find that the manuscript has received many criticism in regard to key issues such as the theoretical-methodological assumptions of the study and the interpretation of the data. Accordingly, one of the reviewers has suggested its immediate rejection, while the other asks for a major set of (extensive) revisions.

Hence, I would like to leave to the authors the possibility of revising the manuscript, in order to send it to the reviewers for their reconsideration, if you decide to submit it.

We would appreciate receiving your revised manuscript by Jun 13 2020 11:59PM. When you are ready to submit your revision, log on to <https://www.editorialmanager.com/pone/> and select the 'Submissions Needing Revision' folder to locate your manuscript file.

If you would like to make changes to your financial disclosure, please include your updated statement in your cover letter.

To enhance the reproducibility of your results, we recommend that if applicable you deposit your laboratory protocols in [protocols.io](http://protocols.io), where a protocol can be assigned its own identifier (DOI) such that it can be cited independently in the future. For instructions see: <http://journals.plos.org/plosone/s/submission-guidelines#loc-laboratory-protocols>

Please include the following items when submitting your revised manuscript:

- A rebuttal letter that responds to each point raised by the academic editor and reviewer(s). This letter should be uploaded as separate file and labeled 'Response to Reviewers'.
- A marked-up copy of your manuscript that highlights changes made to the original version. This file should be uploaded as separate file and labeled 'Revised Manuscript with Track Changes'.
- An unmarked version of your revised paper without tracked changes. This file should be uploaded as separate file and labeled 'Manuscript'.

Please note while forming your response, if your article is accepted, you may have the opportunity to make the peer review history publicly available. The record will include editor decision letters (with reviews) and your responses to reviewer comments. If eligible, we will contact you to opt in or out.

We look forward to receiving your revised manuscript.

Kind regards,

Sergio A. Useche, Ph.D.
Academic Editor
PLOS ONE

Journal Requirements:

When submitting your revision, we need you to address these additional requirements.

1. Please ensure that your manuscript meets PLOS ONE's style requirements, including those for file naming. The PLOS ONE style templates can be found at

<https://journals.plos.org/plosone/s/file?id=wjVg/PLOSOne_formatting_sample_main_body.pdf> and

<https://journals.plos.org/plosone/s/file?id=ba62/PLOSOne_formatting_sample_title_authors_affiliations.pdf>

2. We noticed you have some minor occurrence of overlapping text with the following previous publication(s), which needs to be addressed:

<https://doi.org/10.1016/j.sbspro.2015.04.122>

In your revision ensure you cite all your sources (including your own works), and quote or rephrase any duplicated text outside the methods section. Further consideration is dependent on these concerns being addressed.

 3. We note that you have indicated that data from this study are available upon request. PLOS only allows data to be available upon request if there are legal or ethical restrictions on sharing data publicly. For more information on unacceptable data access restrictions, please see <http://journals.plos.org/plosone/s/data-availability#loc-unacceptable-data-access-restrictions>.

In your revised cover letter, please address the following prompts:

a) If there are ethical or legal restrictions on sharing a de-identified data set, please explain them in detail (e.g., data contain potentially sensitive information, data are owned by a third-party organization, etc.) and who has imposed them (e.g., an ethics committee). Please also provide contact information for a data access committee, ethics committee, or other institutional body to which data requests may be sent.

b) If there are no restrictions, please upload the minimal anonymized data set necessary to replicate your study findings as either Supporting Information files or to a stable, public repository and provide us with the relevant URLs, DOIs, or accession numbers. For a list of acceptable repositories, please see <http://journals.plos.org/plosone/s/data-availability#loc-recommended-repositories>.

We will update your Data Availability statement on your behalf to reflect the information you provide.

We published SPSS and M+ datasets and syntaxes related with the study on Open Access Figshare repository: https://doi.org/10.6084/m9.figshare.12357593.v1

4. Thank you for stating the following financial disclosure:

"The funders had no role in study design, data collection and analysis, decision to
publish, or preparation of the manuscript."

At this time, please address the following queries:

a. Please clarify the sources of funding (financial or material support) for your study. List the grants or organizations that supported your study, including funding received from your institution.
b. State what role the funders took in the study. If the funders had no role in your study, please state: “The funders had no role in study design, data collection and analysis, decision to publish, or preparation of the manuscript.”
c. If any authors received a salary from any of your funders, please state which authors and which funders.
d. If you did not receive any funding for this study, please state: “The authors received no specific funding for this work.”

Please include your amended statements within your cover letter; we will change the online submission form on your behalf.

Reviewers' comments:

Reviewer's Responses to Questions

**Comments to the Author**

1. Is the manuscript technically sound, and do the data support the conclusions?

The manuscript must describe a technically sound piece of scientific research with data that supports the conclusions. Experiments must have been conducted rigorously, with appropriate controls, replication, and sample sizes. The conclusions must be drawn appropriately based on the data presented.

Reviewer #1: Yes

Reviewer #2: No

2. Has the statistical analysis been performed appropriately and rigorously?

Reviewer #1: I Don't Know

Reviewer #2: N/A

3. Have the authors made all data underlying the findings in their manuscript fully available?

The [PLOS Data policy](http://www.plosone.org/static/policies.action#sharing) requires authors to make all data underlying the findings described in their manuscript fully available without restriction, with rare exception (please refer to the Data Availability Statement in the manuscript PDF file). The data should be provided as part of the manuscript or its supporting information, or deposited to a public repository. For example, in addition to summary statistics, the data points behind means, medians and variance measures should be available. If there are restrictions on publicly sharing data—e.g. participant privacy or use of data from a third party—those must be specified.

Reviewer #1: No

Reviewer #2: No

4. Is the manuscript presented in an intelligible fashion and written in standard English?

PLOS ONE does not copyedit accepted manuscripts, so the language in submitted articles must be clear, correct, and unambiguous. Any typographical or grammatical errors should be corrected at revision, so please note any specific errors here.

Reviewer #1: No

Reviewer #2: No

5. Review Comments to the Author

Please use the space provided to explain your answers to the questions above. You may also include additional comments for the author, including concerns about dual publication, research ethics, or publication ethics. (Please upload your review as an attachment if it exceeds 20,000 characters)

Reviewer #1: The heart of this article is a good experiment, which is certainly worthy of publication. There are several flaws that mean that the article will require substantial revision before this should occur.

First, the standard of English is not adequate, though it is close. Most paragraphs had at least one phrase which suggested it was written by a non-native speaker and which interfered with swift interpretation. At times, I was unable to be sure I understood the authors' intended meaning. Due to the number of errors, I have not attempted to correct them, but suggest the authors engage a professional editor.

Second, the authors have assumed that the Brexit vote led to negative changes in attitudes toward Polish workers, and that this led to a range of negative psychological consequences.

I thought one of the most interesting findings was the extent to which the Brexit vote did not lead to negative changes in attitudes toward Polish workers. The proportion reporting a negative change for their supervisors was only two percentage points higher than reporting a positive change, and both were low. No statistical testing was conducted, and it isn't immediately obvious what sort would be most appropriate. Indeed, this might depend on what one intended for the results. Looking at this result as a measure of how British society has reacted to the vote suggests that it hasn't changed the perspectives of supervisors much at all. The relatively small number of respondents who reported any change in either direction is notable, and the bipolarity of this movement is notable. On the face of it, this looks like a null hypothesis to me, since one might expect that at least a small proportion of people would report a change even if there were not one. One might also expect that some people would assume the direction would be negative, and that this might account for the small difference.

The difference was more marked in the change in the attitudes of coworkers, but still not overwhelming.

On the whole, these results are worth discussing, not least in the context of the literature which argues that right-wing anti-globalisation will increase stress for minorities (see eg Macgregor-Bowles and Bowles, 2017. Trump, Brexit, Right-wing Anti-globalisation, and An Uncertain Future for Public Health). In short, the authors should integrate their findings with the social science literature beyond psychology, especially in a multi-disciplinary journal like PLOS ONE.

The difference in the apparent reactions to Brexit from co-workers and supervisors is especially interesting. It could be due to several factors. Most basically, people tend to have more co-workers than supervisors, so there are more opportunities to perceive a negative change, and this is important when most people don't perceive any change at all. Additionally, supervisors may feel less threatened by Polish workers as they do not represent such intense competition for jobs (and may represent a pool of relatively cheap, but highly skilled labour). Alternatively, supervisors may operate in social circles in which xenophobia is more sharply condemned than their workers. Another possibility is that supervisors may also be aware of their positions of authority and the need to moderate their behaviour or could more strictly constrain their behaviour. Alternatively, they may simply be more attuned to the possibility that their Polish workers may be at risk of feeling marginalised following the Brexit vote. Assessing the results by geographic area of the respondent would be interesting.

|  | Coworkers | Supervisor |
| --- | --- | --- |
| South England | 2.98 | 2.91 |
| Central England | 3.13 | 3.07 |
| North England | 3.14 | 3.03 |
| South Scotland | 3 | 3 |
| Central Scotland | 3.23 | 3.08 |
| North Scotland | 2.93 | 3.07 |
| North Ireland | 3.2 | 3.16 |
| Wales | 3.22 | 3.06 |

| **ANOVA** | | | | | | |
| --- | --- | --- | --- | --- | --- | --- |
|  | | Sum of Squares | df | Mean Square | F | Sig. |
| Coworkers | Between Groups | 4,949 | 7 | ,707 | 2,459 | ,017 |
|  | Within Groups | 155,276 | 540 | ,288 |  |  |
|  | Total | 160,224 | 547 |  |  |  |
| Supervisors | Between Groups | 3,448 | 7 | ,493 | 2,078 | ,044 |
|  | Within Groups | 127,535 | 538 | ,237 |  |  |
|  | Total | 130,984 | 545 |  |  |  |

| **Multiple Comparisons** | | | | | | | |
| --- | --- | --- | --- | --- | --- | --- | --- |
| Bonferroni | | | | | | | |
| Dependent Variable | (I) Region Miejsce zamieszkania w UK | (J) Region Miejsce zamieszkania w UK | Mean Difference (I-J) | Std. Error | Sig. | 95% Confidence Interval | |
|  |  |  |  |  |  | Lower Bound | Upper Bound |
| Coworkers | South England | Central England | -,146 | ,058 | ,333 | -,33 | ,04 |
|  |  | North England | -,156 | ,076 | 1,000 | -,39 | ,08 |
|  |  | South Scotland | -,018 | ,193 | 1,000 | -,62 | ,59 |
|  |  | Central Scotland | -,249 | ,093 | ,217 | -,54 | ,04 |
|  |  | North Scotland | ,054 | ,107 | 1,000 | -,28 | ,39 |
|  |  | North Ireland | -,218 | ,113 | 1,000 | -,57 | ,14 |
|  |  | Wales | -,240 | ,131 | 1,000 | -,65 | ,17 |
|  | Central England | South England | ,146 | ,058 | ,333 | -,04 | ,33 |
|  |  | North England | -,011 | ,080 | 1,000 | -,26 | ,24 |
|  |  | South Scotland | ,128 | ,195 | 1,000 | -,48 | ,74 |
|  |  | Central Scotland | -,103 | ,097 | 1,000 | -,41 | ,20 |
|  |  | North Scotland | ,199 | ,111 | 1,000 | -,15 | ,55 |
|  |  | North Ireland | -,072 | ,116 | 1,000 | -,44 | ,29 |
|  |  | Wales | -,095 | ,134 | 1,000 | -,52 | ,33 |
|  | North England | South England | ,156 | ,076 | 1,000 | -,08 | ,39 |
|  |  | Central England | ,011 | ,080 | 1,000 | -,24 | ,26 |
|  |  | South Scotland | ,138 | ,201 | 1,000 | -,49 | ,77 |
|  |  | Central Scotland | -,092 | ,109 | 1,000 | -,43 | ,25 |
|  |  | North Scotland | ,210 | ,121 | 1,000 | -,17 | ,59 |
|  |  | North Ireland | -,062 | ,126 | 1,000 | -,46 | ,33 |
|  |  | Wales | -,084 | ,143 | 1,000 | -,53 | ,36 |
|  | South Scotland | South England | ,018 | ,193 | 1,000 | -,59 | ,62 |
|  |  | Central England | -,128 | ,195 | 1,000 | -,74 | ,48 |
|  |  | North England | -,138 | ,201 | 1,000 | -,77 | ,49 |
|  |  | Central Scotland | -,231 | ,208 | 1,000 | -,88 | ,42 |
|  |  | North Scotland | ,071 | ,215 | 1,000 | -,60 | ,75 |
|  |  | North Ireland | -,200 | ,218 | 1,000 | -,88 | ,48 |
|  |  | Wales | -,222 | ,228 | 1,000 | -,94 | ,49 |
|  | Central Scotland | South England | ,249 | ,093 | ,217 | -,04 | ,54 |
|  |  | Central England | ,103 | ,097 | 1,000 | -,20 | ,41 |
|  |  | North England | ,092 | ,109 | 1,000 | -,25 | ,43 |
|  |  | South Scotland | ,231 | ,208 | 1,000 | -,42 | ,88 |
|  |  | North Scotland | ,302 | ,133 | ,652 | -,11 | ,72 |
|  |  | North Ireland | ,031 | ,137 | 1,000 | -,40 | ,46 |
|  |  | Wales | ,009 | ,153 | 1,000 | -,47 | ,49 |
|  | North Scotland | South England | -,054 | ,107 | 1,000 | -,39 | ,28 |
|  |  | Central England | -,199 | ,111 | 1,000 | -,55 | ,15 |
|  |  | North England | -,210 | ,121 | 1,000 | -,59 | ,17 |
|  |  | South Scotland | -,071 | ,215 | 1,000 | -,75 | ,60 |
|  |  | Central Scotland | -,302 | ,133 | ,652 | -,72 | ,11 |
|  |  | North Ireland | -,271 | ,148 | 1,000 | -,73 | ,19 |
|  |  | Wales | -,294 | ,162 | 1,000 | -,80 | ,21 |
|  | North Ireland | South England | ,218 | ,113 | 1,000 | -,14 | ,57 |
|  |  | Central England | ,072 | ,116 | 1,000 | -,29 | ,44 |
|  |  | North England | ,062 | ,126 | 1,000 | -,33 | ,46 |
|  |  | South Scotland | ,200 | ,218 | 1,000 | -,48 | ,88 |
|  |  | Central Scotland | -,031 | ,137 | 1,000 | -,46 | ,40 |
|  |  | North Scotland | ,271 | ,148 | 1,000 | -,19 | ,73 |
|  |  | Wales | -,022 | ,166 | 1,000 | -,54 | ,50 |
|  | Wales | South England | ,240 | ,131 | 1,000 | -,17 | ,65 |
|  |  | Central England | ,095 | ,134 | 1,000 | -,33 | ,52 |
|  |  | North England | ,084 | ,143 | 1,000 | -,36 | ,53 |
|  |  | South Scotland | ,222 | ,228 | 1,000 | -,49 | ,94 |
|  |  | Central Scotland | -,009 | ,153 | 1,000 | -,49 | ,47 |
|  |  | North Scotland | ,294 | ,162 | 1,000 | -,21 | ,80 |
|  |  | North Ireland | ,022 | ,166 | 1,000 | -,50 | ,54 |
| Supervisors | South England | Central England | -,156 | ,052 | ,084 | -,32 | ,01 |
|  |  | North England | -,116 | ,069 | 1,000 | -,33 | ,10 |
|  |  | South Scotland | -,085 | ,175 | 1,000 | -,64 | ,46 |
|  |  | Central Scotland | -,164 | ,085 | 1,000 | -,43 | ,10 |
|  |  | North Scotland | -,157 | ,098 | 1,000 | -,46 | ,15 |
|  |  | North Ireland | -,245 | ,103 | ,484 | -,57 | ,08 |
|  |  | Wales | -,141 | ,119 | 1,000 | -,52 | ,23 |
|  | Central England | South England | ,156 | ,052 | ,084 | -,01 | ,32 |
|  |  | North England | ,040 | ,073 | 1,000 | -,19 | ,27 |
|  |  | South Scotland | ,071 | ,177 | 1,000 | -,48 | ,63 |
|  |  | Central Scotland | -,008 | ,089 | 1,000 | -,29 | ,27 |
|  |  | North Scotland | -,001 | ,101 | 1,000 | -,32 | ,32 |
|  |  | North Ireland | -,089 | ,106 | 1,000 | -,42 | ,24 |
|  |  | Wales | ,015 | ,122 | 1,000 | -,37 | ,40 |
|  | North England | South England | ,116 | ,069 | 1,000 | -,10 | ,33 |
|  |  | Central England | -,040 | ,073 | 1,000 | -,27 | ,19 |
|  |  | South Scotland | ,031 | ,182 | 1,000 | -,54 | ,60 |
|  |  | Central Scotland | -,048 | ,099 | 1,000 | -,36 | ,26 |
|  |  | North Scotland | -,041 | ,110 | 1,000 | -,39 | ,30 |
|  |  | North Ireland | -,129 | ,115 | 1,000 | -,49 | ,23 |
|  |  | Wales | -,025 | ,130 | 1,000 | -,43 | ,38 |
|  | South Scotland | South England | ,085 | ,175 | 1,000 | -,46 | ,64 |
|  |  | Central England | -,071 | ,177 | 1,000 | -,63 | ,48 |
|  |  | North England | -,031 | ,182 | 1,000 | -,60 | ,54 |
|  |  | Central Scotland | -,079 | ,189 | 1,000 | -,67 | ,52 |
|  |  | North Scotland | -,071 | ,195 | 1,000 | -,68 | ,54 |
|  |  | North Ireland | -,160 | ,198 | 1,000 | -,78 | ,46 |
|  |  | Wales | -,056 | ,207 | 1,000 | -,71 | ,59 |
|  | Central Scotland | South England | ,164 | ,085 | 1,000 | -,10 | ,43 |
|  |  | Central England | ,008 | ,089 | 1,000 | -,27 | ,29 |
|  |  | North England | ,048 | ,099 | 1,000 | -,26 | ,36 |
|  |  | South Scotland | ,079 | ,189 | 1,000 | -,52 | ,67 |
|  |  | North Scotland | ,008 | ,121 | 1,000 | -,37 | ,39 |
|  |  | North Ireland | -,081 | ,125 | 1,000 | -,47 | ,31 |
|  |  | Wales | ,023 | ,139 | 1,000 | -,41 | ,46 |
|  | North Scotland | South England | ,157 | ,098 | 1,000 | -,15 | ,46 |
|  |  | Central England | ,001 | ,101 | 1,000 | -,32 | ,32 |
|  |  | North England | ,041 | ,110 | 1,000 | -,30 | ,39 |
|  |  | South Scotland | ,071 | ,195 | 1,000 | -,54 | ,68 |
|  |  | Central Scotland | -,008 | ,121 | 1,000 | -,39 | ,37 |
|  |  | North Ireland | -,089 | ,134 | 1,000 | -,51 | ,33 |
|  |  | Wales | ,016 | ,147 | 1,000 | -,45 | ,48 |
|  | North Ireland | South England | ,245 | ,103 | ,484 | -,08 | ,57 |
|  |  | Central England | ,089 | ,106 | 1,000 | -,24 | ,42 |
|  |  | North England | ,129 | ,115 | 1,000 | -,23 | ,49 |
|  |  | South Scotland | ,160 | ,198 | 1,000 | -,46 | ,78 |
|  |  | Central Scotland | ,081 | ,125 | 1,000 | -,31 | ,47 |
|  |  | North Scotland | ,089 | ,134 | 1,000 | -,33 | ,51 |
|  |  | Wales | ,104 | ,151 | 1,000 | -,37 | ,58 |
|  | Wales | South England | ,141 | ,119 | 1,000 | -,23 | ,52 |
|  |  | Central England | -,015 | ,122 | 1,000 | -,40 | ,37 |
|  |  | North England | ,025 | ,130 | 1,000 | -,38 | ,43 |
|  |  | South Scotland | ,056 | ,207 | 1,000 | -,59 | ,71 |
|  |  | Central Scotland | -,023 | ,139 | 1,000 | -,46 | ,41 |
|  |  | North Scotland | -,016 | ,147 | 1,000 | -,48 | ,45 |
|  |  | North Ireland | -,104 | ,151 | 1,000 | -,58 | ,37 |

We thank the reviewer for this interesting observation and conclusion. We applied this in the discussion.


Third, in setting up and interpreting the experiment, the authors have assumed the direction of the causal arrow. They assume that Poles who say that they have experienced a negative change in the attitude or behaviour of supervisors have actually experienced this. While this is almost certainly true in some cases, it should not be assumed for all cases. It might be that people who experience elevated levels of perceived stress or decreased levels of wellbeing are more likely to interpret the words or actions of their supervisors as discriminatory. If Brexit has added to their stress due to feeling generally unwanted by the UK, or uncertainty about their futures, then it could be that they will project this general feeling onto the people in their lives such as coworkers and supervisors.

Figure 1. Structural Equation Model of the effect of after vote NCS and NCC. RMSEA = .000 CFI = 1.00.

Due to cross-sectional nature of the study, we had tested the alternative model with perceived stress and well-being being independent variables in the casual model. The model has been identified but there are no statistically significant relationships. Thus, we cannot empirically assume that people who experience elevated levels of perceived stress or decreased levels of wellbeing are more likely to interpret the words or actions of their supervisors as discriminatory and at the same time, report it in our study.

It might also be that people whose stress was increased by Brexit began to perform less well at work, leading to comments or behaviours that they then interpreted as discriminatory. It is plausible that these pathways account for a reasonable amount of the observed correlations, and they need to be discussed in the paper.

We didn’t include such mediator as job performance in our study, thus we cannot test the hypothesis proposed by the reviewer of mediating role of job performance between perceived stress and perceived negative behaviour. However, we do agree that in some cases this relationship might be possible.

The authors drew a parallel between intention to leave a work place and intention to leave the UK. While there may be some parallels, they are clearly different. There is a vast literature on immigration between countries, and this should be used to inform and support the authors' analysis.

This is another area where the causal arrow might point two directions. Perhaps those already intending to leave the UK perceive more stress. Moving countries is stressful! It is also possible that those intending to leave the UK perceive discrimination more readily as confirmation bias. This also needs discussion.

Figure 2. Structural Equation Model of the effect of after vote NCS and NCC. RMSEA = .231 CFI = .650.

We also estimated the model with intention to leave the UK being independent variable for negative attitude change of coworkers and supervisor with mediating role of perceived stress and well-being. The goodness-of-fit indexes are not supporting the model. Thus, the hypothesis of the reviewer that intending to leave the UK perceive more stress and then result in perceived negative attitude changes.

Fourth, a little more information about the methods would be useful. Specifically, how was it confirmed that Polish participants had British supervisors, and that they were talking about British coworkers (one can imagine that many had coworkers from the UK and a variety of other countries)?

We measures this using two questions indicating the possible change in behaviour or attitude of their British coworkers or supervisors:

*Have you noticed a change in the attitude/behaviour of your British co-workers towards you after the Brexit referendum?*

- *I have seen a very positive change in the behaviour of British coworkers*
- *I have seen a positive change in the attitude/behaviour of British coworkers*
- *I have not noticed any change in the attitude/behaviour of British coworkers*
- *I have seen a negative change in the attitude/behaviour of British coworkers*
- *I have seen a very negative change in the attitude/behaviour of British coworkers*

*Have you noticed a change in the attitude/behaviour of your British supervisors towards you after the Brexit referendum?*

- *I have seen a very positive change in the attitude/behaviour of British supervisors*
- *I have seen a positive change in the attitude/behaviour of British supervisors*
- *I have not noticed any change in the attitude/behaviour of British supervisors*
- *I have seen a negative change in the attitude/behaviour of British supervisors*
- *I have seen a very negative change in the attitude/behaviour of British supervisors*

Additionally, how did the authors ensure that participants had been in their jobs long enough to make good observations about changes in their supervisor and coworkers?

Unfortunately, we haven’t measured the work tenue. However, we were interested in the subjective perception of the changes of participants regardless the employment length. We agree with the reviewer it would be interesting to see if work tenue have any relationship with change in behaviour observations.

This relates to a fifth issue, which is that the authors assume that the Brexit vote would be a clear turning point for an increase in racist behaviour. However, the lead up to the Brexit vote, with its rhetoric around immigrants, probably means that the Brexit vote itself was not a really decisive break with the preceding months. Those looking for an excuse to be racist could seek cover in the need to debate Brexit. On the whole, this might mean that the results, by relying on a comparison between the Brexit vote and the months before the Brexit vote, could underestimate the overall effect of the rise of anti-immigration sentiment on Polish workers in the UK.

Sixth, the authors could consider whether social support moderated the effect of changes in coworkers but not supervisors because coworkers are in fact a primary pool of potential social supports for immigrants. Therefore, if you coworkers are treating you less well, one of your primary potential sources of social support is no longer available.

We thank the reviewer for this interesting observation and conclusion. We applied this in the discussion.

In general, the statistical testing looked reasonable. Some of the modelling was beyond my capacity to judge, though I note that it might rely on the causal arrow pointing only one way, which seems inappropriate given what I've written above.

Reviewer #2: Thank you for your submission. The submission does not seem to have a substantive paper. Thank you for your submission. The submission does not seem to have a substantive paper. The result is not significant.
